# A dual-action humanized DKK-1 antibody that activates the Wnt/β-catenin signaling pathway and inhibits the RANKL signaling pathway for osteoporosis therapy

**DOI:** 10.3389/fimmu.2026.1906434

**Published:** 2026-08-27

**Authors:** Sheng Hou, Tianyu Gao, Yimei Wu, Hao Wang, Dapeng Zhang, Qingcheng Guo, Jin Xu, Huaizu Guo, Weizhu Qian

**Affiliations:** 1 Taizhou Mabtech Pharmaceuticals Co., Ltd, Taizhou, China; 2 State key laboratory of macromolecular drugs and large-scale preparation, NMPA Key Laboratory for Quality Control of Therapeutic Monoclonal Antibodies, Shanghai Zhangjiang Biotechnology Co., Ltd, Shanghai, China; 3 State key laboratory of macromolecular drugs and large-scale preparation, School of Pharmaceutical Sciences and Food Engineering, Liaocheng University, Liaocheng, China; 4 State key laboratory of macromolecular drugs and large-scale preparation, School of Pharmaceutical Sciences, Wenzhou Medical University, Wenzhou, China

**Keywords:** DKK-1 antibody, humanization, OPG-RANKL-RANK signaling pathway, osteogenesis-osteoclastogenesis Transwell co-culture, osteoporosis, Wnt/β-catenin signaling pathway

## Abstract

**Introduction:**

Osteoporosis remains a major global health challenge, and current single-pathway therapies often fail to achieve coordinated bone remodeling. Dickkopf-1 (DKK-1) is a potent inhibitor of the Wnt/β-catenin signaling pathway and plays a critical role in osteoporotic bone loss. Neutralization of DKK-1 represents a promising therapeutic strategy to promote bone formation while simultaneously inhibiting bone resorption.

**Methods:**

A humanized anti-DKK-1 monoclonal antibody (IgG4) was generated via CDR grafting combined with structure-guided back-mutations. Its binding affinity and specificity were assessed by ELISA and surface plasmon resonance. The antibody's ability to restore Wnt/β-catenin signaling was evaluated in human bone marrow mesenchymal stem cells (hMSCs) by Western blotting. Osteogenic differentiation and mineralization were assessed by ALP activity assay and Alizarin Red S staining, respectively. An osteoblast-osteoclast Transwell co-culture model was established to evaluate the antibody's effects on osteoclastogenesis via the OPG/RANKL axis, with osteoclast differentiation assessed by TRAP staining and qPCR. Developability properties, including stability and immunogenicity, were also characterized.

**Results:**

The humanized antibody bound to DKK-1 with high affinity (EC_₅₀_ = 32.8 ng/mL, KD = 2.760 × 10^⁻¹⁰^ mol/L) and showed no cross-reactivity to DKK-2, DKK-3, or DKK-4. In hMSCs, the antibody restored Wnt/β-catenin signaling, as evidenced by nuclear β-catenin accumulation, GSK-3β phosphorylation, and c-Myc upregulation, leading to increased ALP activity, OPG secretion, and mineralization. In the Transwell co-culture model, the antibody restored the OPG/sRANKL molar ratio and significantly suppressed osteoclast marker gene expression (ACP5, CALCR, CTSK, ITGB3, MMP9, and NFATC1) as well as TRAP-positive multinucleated cell formation (from 29 ± 5 per field in the undifferentiated control to 2 ± 1 per field in the antibody-treated group). The antibody also exhibited favorable developability properties, including long-term stability at 2 -8°C for over two years and low cellular immunogenicity.

**Discussion:**

These findings demonstrate that the humanized anti-DKK-1 antibody exerts dual anabolic and anti-resorptive effects through the Wnt/β-catenin/OPG/RANKL axis. This dual mechanism of action positions the antibody as a promising therapeutic candidate for osteoporosis, warranting further preclinical and clinical evaluation.

## Introduction

Osteoporosis is a prevalent skeletal disorder characterized by progressive bone loss and microarchitectural deterioration, leading to increased fracture risk and substantial morbidity (1). Most current therapies act predominantly on either bone resorption or bone formation: antiresorptive agents such as bisphosphonates and denosumab reduce osteoclast activity, while anabolic agents such as teriparatide and romosozumab stimulate osteoblast function (2–4). However, the pathophysiology of osteoporosis involves an uncoupling of bone remodeling, with excessive osteoclast-mediated resorption and inadequate osteoblast-mediated formation occurring simultaneously. A single agent capable of addressing both processes would meet a significant unmet clinical need.

The Wnt/β-catenin signaling pathway is a central regulator of bone mass (5–7). Activation of canonical Wnt signaling promotes osteoblast differentiation from mesenchymal precursors, enhances osteoblast survival, and indirectly suppresses osteoclastogenesis by modulating the expression of key paracrine factors (8, 9). A critical downstream mediator of this indirect effect is osteoprotegerin (OPG), a soluble decoy receptor secreted by cells of the osteoblast lineage. OPG binds receptor activator of nuclear factor-κB ligand (RANKL) with high affinity, preventing RANKL from engaging its receptor RANK on osteoclast precursors, thereby inhibiting osteoclast differentiation (10). Multiple studies have confirmed that Wnt/β-catenin signaling upregulates OPG expression, establishing a direct mechanistic link between osteoblast activation and the suppression of bone resorption (8, 11, 12). Thus, reinforcing this endogenous OPG/RANKL regulatory axis via Wnt activation represents an attractive therapeutic strategy (13).

Dickkopf-1 (DKK-1) is a potent secreted antagonist of the Wnt/β-catenin pathway (14, 15). It binds to the co-receptors LRP5/6 and Kremen, triggering internalization of the Wnt receptor complex and blockade of downstream signaling (16). DKK-1 expression is elevated in pathological bone loss conditions, including estrogen deficiency, glucocorticoid excess, and multiple myeloma; its overexpression in mice results in osteopenia. Conversely, DKK-1 deficiency or neutralization leads to increased bone mass (17). Consequently, DKK-1 has emerged as a promising molecular target for promoting bone formation while indirectly inhibiting bone resorption via OPG (18).

**Figure 1:**
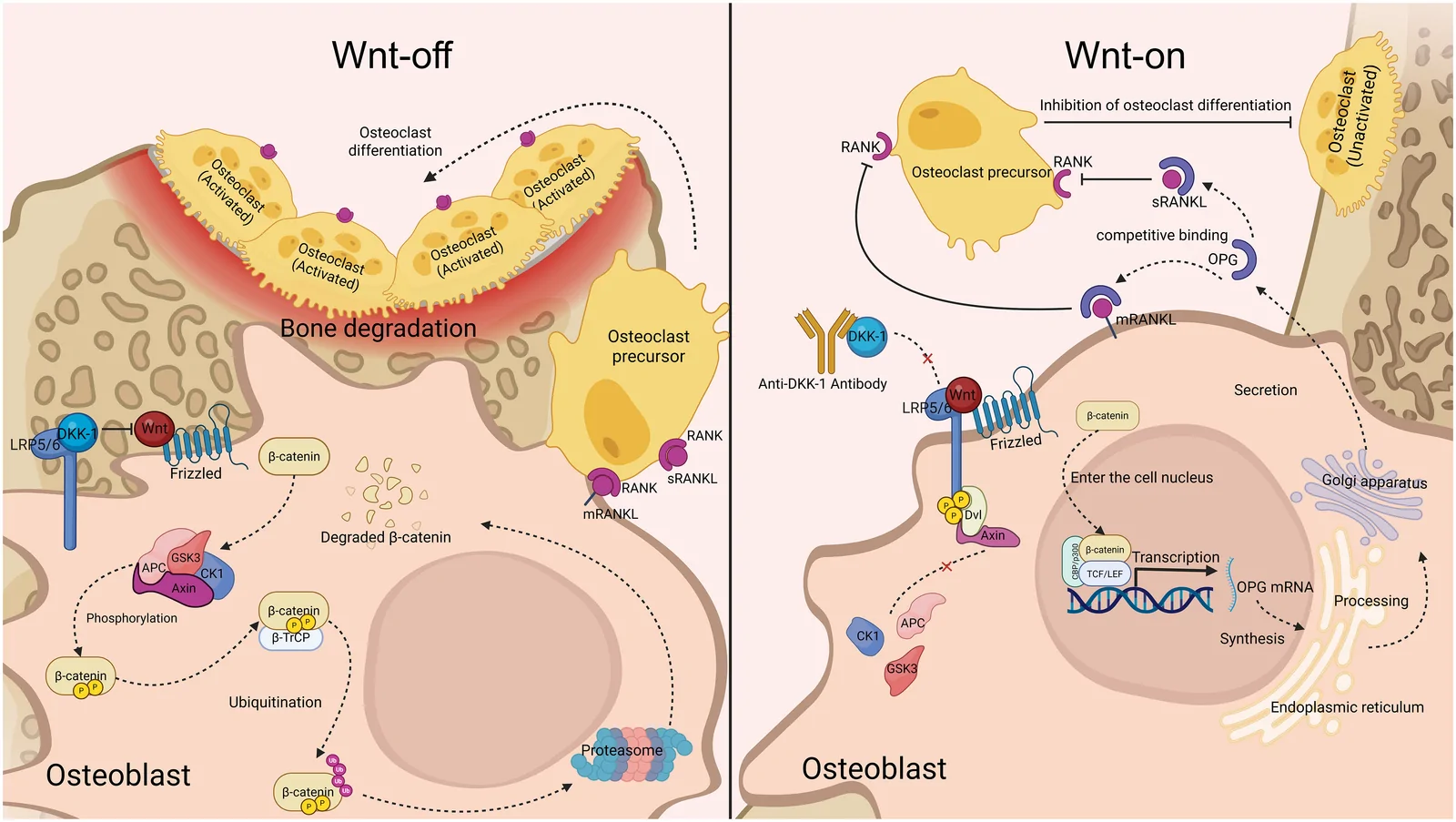
The DKK-1 antibody regulates osteoclast differentiation through the Wnt/β-catenin-OPG-RANKL-RANK pathway.

Recently, our previous study demonstrated that this humanized anti-DKK-1 monoclonal antibody not only increases bone mass and strength but also improves muscle performance in orchiectomized mice, with sequential alendronate treatment producing additive benefits (19). In the present study, we further investigate the underlying signaling mechanisms of this antibody (**Figure 1**). The murine antibody was humanized by CDR grafting with structure-guided back-mutations, and an IgG4 constant region was chosen to minimize Fc-mediated effector functions (20, 21). The antibody exhibited high-affinity binding and potent functional activity. To assess the antibody’s capacity to simultaneously regulate bone formation and resorption, we employed a human osteoblast–osteoclast Transwell co-culture system that permits paracrine crosstalk analysis (22–24). Our results demonstrate that the humanized antibody neutralizes DKK-1, restores Wnt/β-catenin signaling, promotes osteogenic differentiation, and markedly upregulates OPG secretion. The elevated OPG effectively antagonizes RANKL, leading to substantial suppression of osteoclastogenic gene expression and osteoclast differentiation (25–27). These findings establish that this anti-DKK-1 antibody acts through the Wnt/β-catenin/OPG axis as a dual-action agent with both anabolic and anti-resorptive properties, supporting its further development as a potential therapeutic candidate for osteoporosis.

## Materials and methods

### Materials

#### Reagents

Recombinant human DKK-1, mouse-derived anti-DKK-1 antibody, Wnt3a, and RANKL were obtained from Shanghai Sinomab Biotechnology Co., Ltd. (Shanghai, China); recombinant human M-CSF from Yeasen Biotechnology (Shanghai) Co., Ltd.; Ficoll-Paque Plus (1.077 g/mL), and human DKK-2, DKK-3, and DKK-4 recombinant proteins from Beijing Solarbio Science & Technology Co., Ltd; RIPA Lysis Buffer (Strong), PMSF Solution (100 mmol/L), BeyoEmbryo™ 0.1% Gelatin Solution, Alkaline Phosphatase Assay Kit, Hematoxylin Staining Solution, and BCA Protein Assay Kit (P0012) were acquired from Beyotime Biotech Inc. (Shanghai, China); Transwell^®^ inserts (24 mm diameter, 0.4 μm pore size, polyester membrane) were purchased from Corning Incorporated (Corning, NY, USA);β-Glycerophosphate disodium salt hydrate (BGP), L-ascorbic acid, and dexamethasone were sourced from Merck KGaA (Darmstadt, Germany); The Cell Counting Kit-8 (CCK-8) was obtained from GlpBio (Montclair, CA, USA); the Alizarin Red S Staining Kit from Beijing Baiaolaibo Technology Co., Ltd.; the TRAP Staining Kit from Wuhan ABclonal Biotechnology Co., Ltd.; The Reverse Transcription System from Promega Corporation (Madison, WI, USA); DNA primers were synthesized by Tsingke Biotechnology Co., Ltd. (Beijing, China); the IFN-γ and IL-2 ELISPOT kits, along with the HRP-conjugated goat anti-human IgG-Fc secondary antibody, were obtained from Sino Biological Inc. (Beijing, China); Cell culture media (DMEM and α-MEM), fetal bovine serum (FBS), and penicillin/streptomycin were purchased from Gibco™ (Thermo Fisher Scientific, Waltham, MA, USA), along with TRIzol™ Reagent, Power SYBR™ Green PCR Master Mix, Human sRANKL (Soluble) ELISA Kit, and NE-PER™ Nuclear and Cytoplasmic Extraction Reagents; The Human OPG (Osteoprotegerin) ELISA Kit was purchased from Elabscience Biotechnology Co., Ltd. (Wuhan, China) and antibodies against β-catenin, c-Myc, Histone H3, p-GSK-3β (Ser9), total GSK-3β, and GAPDH were all obtained from Cell Signaling Technology (Danvers, MA, USA).

#### Equipment

The biosafety cabinet was purchased from AIRTECH (Suzhou, China). The Heracell™ 150i CO_2_ incubator, StepOnePlus Real-Time PCR System, and Fresco 17 microcentrifuge were obtained from Thermo Fisher Scientific (Waltham, MA, USA). Surface plasmon resonance measurements were performed on a Biacore T200 system (GE Healthcare, Chicago, IL, USA; now Cytiva). The Centrifuge 5810R high-speed refrigerated centrifuge was from Eppendorf (Hamburg, Germany). The GloMax^®^ 96 Microplate Luminometer was purchased from Promega Corporation (Madison, WI, USA), and the SpectraMax M5 multi-mode microplate reader from Molecular Devices (Sunnyvale, CA, USA). An Olympus CKX41 inverted microscope (Olympus, Tokyo, Japan) was used for cell observation. ELISPOT assays were evaluated using an AID ELISPOT reader (ELR08IFL, AID GmbH, Germany). Western blot images were captured using a Tanon 4800 chemiluminescence imaging system (Tanon Science & Technology Co., Ltd., Shanghai, China). SEC analysis was carried out on a Waters ACQUITY UPLC H-Class system equipped with a PDA eλ detector and an ACQUITY UPLC Protein BEH SEC column (200 Å, 1.7 μm, 4.6 mm × 150 mm; Waters Corporation, Milford, MA, USA). Differential scanning calorimetry (DSC) was performed using a MicroCal VP-Capillary DSC system (Malvern Panalytical, UK).

#### Cell line

Human bone marrow mesenchymal stem cells (hMSCs) were purchased from the Cell Bank of the Type Culture Collection Committee, Chinese Academy of Sciences (Shanghai, China). Human peripheral blood mononuclear cells (hPBMCs) were isolated from the peripheral blood of healthy donors (n ≥ 5). All donors provided written informed consent, and the study was conducted in accordance with the principles of the Declaration of Helsinki. Saos-2 and HEK-293/TCF-LEF/Luc2p/Hygro cell lines were obtained from Shanghai Sinomab Biotechnology Co., Ltd. (Shanghai, China).

### Methods

#### Transwell co-culture

Human bone marrow mesenchymal stem cells (hMSCs) were seeded at 2.0 × 10^4^ cells per well into the lower chamber of a gelatin-coated 24-well Transwell plate and divided into five groups. The undifferentiated group (Undiff) was maintained in α-MEM supplemented with 10% FBS. The osteogenic induction control group (Control) received osteogenic induction complete medium (α-MEM containing 10% FBS, 50 μg/mL ascorbic acid, 10 mM β-glycerophosphate and 100 nM dexamethasone). The Wnt3a stimulation group (Wnt3a) was treated with osteogenic induction complete medium plus 50 ng/mL Wnt3a. The DKK-1 inhibition group (DKK-1) received osteogenic induction complete medium containing 200 ng/mL DKK-1. The DKK-1 antibody rescue group (hDKK-1 mAb) was cultured in osteogenic induction complete medium supplemented with 200 ng/mL DKK-1 and 10 μg/mL anti-DKK-1 antibody. Half-medium changes were performed every 2–3 days, and osteogenic induction was continued for 12 days. After induction, the lower chamber cells were washed with PBS, and 1 × 10^6^ human peripheral blood mononuclear cells (hPBMCs) were seeded into the upper chamber to initiate co-culture. The medium in each group was replaced as follows: the Control group was switched to co-culture medium (α-MEM containing 5% FBS, 30 ng/mL human M-CSF and 30 ng/mL sRANKL); the Undiff group remained in α-MEM with 10% FBS; the Wnt3a group received co-culture medium plus 50 ng/mL Wnt3a; the DKK-1 group received co-culture medium plus 200 ng/mL DKK-1; and the hDKK-1 mAb group received co-culture medium plus 200 ng/mL DKK-1 and 10 μg/mL anti-DKK-1 antibody. During co-culture, half-medium changes were performed every 2–3 days, and culture was continued for 7 days. Human PBMCs were isolated from at least five independent healthy donors, and hMSCs from at least three independent donors. All experiments were performed with at least three biological replicates (n ≥ 3), each using cells from a different donor. Data are presented as mean ± SD, and all statistical analyses were performed on biological replicates. The consistency of responses across donors was confirmed by comparing the relative trends among different treatment groups.

#### Indirect ELISA method for detecting the binding activity of anti-DKK-1 antibodies

Antigen (10 μg/mL in carbonate coating buffer, pH 9.6) was coated onto ELISA plates (100 μL/well) at 37 °C for 1 h. After blocking with 5% non-fat milk (350 μL/well) at 37 °C for 1.5 h, plates were washed seven times. Serially diluted (three-fold, starting at 10 μg/mL) antibody was added (100 μL/well) and incubated at 37 °C for 1 h. Following seven washes, HRP-conjugated goat anti-human IgG polyclonal secondary antibody (100 μL/well) was added for 1 h at 37 °C. After another seven washes, TMB substrate (50 μL/well) was added, the reaction stopped with 50 μL stop solution, and absorbance read at 450 nm (reference 595 nm).

#### Reporter gene assay for biological activity

HEK-293/TCF-LEF/Luc2p/Hygro cells were detached using trypsin-EDTA (0.02% EDTA-0.05% trypsin), resuspended in α-MEM supplemented with 10% FBS to a density of 1.4 × 10^6^ cells/mL, and seeded at 25 μL per well into 96-well plates. Wnt3a-conditioned medium (100 ng/mL), DKK-1 (250 ng/mL), and two-fold serially diluted anti-DKK-1 antibody (starting from 25 μg/mL) were added at 25 μL each per well. After incubation at 37 °C in an 8% CO_2_ atmosphere for 16 h, 100 μL of Bright-Glo™ reagent was added to each well, and luminescence was measured using a Promega GloMax luminometer.

#### SPR method for studying antibody-antigen interactions

Binding affinity was measured by SPR on a Biacore T200 at 25 °C in HBS-EP buffer (pH 7.4). The antigen was amine-coupled onto a CM5 chip to 50–100 RU. Serial dilutions of the antibody (0.1–100 nM) were injected at 30 μL/min (120 s association, 300 s dissociation). Regeneration used 10 mM glycine (pH 2.5). Sensorgrams were double-referenced, and data were fitted to a 1:1 Langmuir model to obtain ka, kd, and KD. Each sample was analyzed in at least two independent experiments.

#### CCK-8 assay for cell viability

Saos-2 cells were adjusted to a density of 4 × 10^4^ cells/mL, and 100 μL of the cell suspension (containing 4000 cells) was seeded per well in 96-well plates. To minimize edge effects, wells at the plate periphery were filled with PBS and not used for experimental measurements. After 24 h, cells were treated with anti-DKK-1 or isotype IgG (0.01–20 μg/mL) for 24 or 48 h. Controls included negative (complete medium), positive (5% DMSO), and blank (no cells). CCK-8 solution (20 μL/well) was added and incubated for 1.5 h in the dark. Absorbance was read at 450 nm (reference 650 nm), and cell viability was calculated as [(OD_exp − OD_blank)/(OD_neg − OD_blank)] × 100%.

#### ELISPOT assay

Cellular immunogenicity was evaluated using IFN-γ and IL-2 ELISPOT kits. PBMCs (1.5 × 10^5^ cells/well) were seeded onto PVDF plates pre-coated with capture antibodies and stimulated with the humanized anti-DKK-1 antibody, the murine anti-DKK-1 antibody, or a human IgG4 isotype control (all at 10 μg/mL). Anti-CD3 antibody served as the positive control, and unstimulated cells served as the negative control. The plates were incubated at 37 °C for 18–24 h. After cell lysis with ice-cold water, the plates were washed, and biotin-conjugated detection antibody (diluted 1:370) was added for 2 h, followed by streptavidin-alkaline phosphatase (diluted 1:170) for 1 h. Spots were developed with BCIP/NBT substrate and counted using an ELISPOT reader. Each condition was tested in triplicate using PBMCs from at least three independent donors. Data are expressed as spot-forming cells (SFC) per 1.5 × 10^5^ PBMCs.

#### Western blot analysis

Whole-cell lysates were prepared using RIPA buffer supplemented with protease and phosphatase inhibitors. Nuclear and cytoplasmic proteins were extracted using a commercial nuclear-cytoplasmic extraction kit according to the manufacturer’s protocol. Protein concentrations were determined by BCA assay. Equal amounts of protein (20–30 μg) were separated by SDS-PAGE, transferred to PVDF membranes, and blocked with 5% BSA in TBST. Membranes were incubated overnight at 4 °C with primary antibodies against β-catenin, c-Myc, Histone H3, p-GSK-3β (Ser9), total GSK-3β, and GAPDH. After washing, membranes were incubated with HRP-conjugated secondary antibodies, and signals were visualized using ECL substrate. Band intensities were quantified using ImageJ software. GAPDH and Histone H3 served as loading controls for cytoplasmic and nuclear fractions, respectively. All experiments were independently repeated at least three times.

#### Size-exclusion chromatography (SEC)

SEC analysis was performed on a Waters ACQUITY UPLC H-Class system equipped with a PDA eλ detector and an ACQUITY UPLC Protein BEH SEC column (200 Å, 1.7 μm, 4.6 mm × 150 mm). The mobile phase consisted of 10.4 g/L NaH_2_PO_4_·2H_2_O and 47.7 g/L Na_2_HPO_4_·12H_2_O, pH 7.0, filtered through a 0.22 μm membrane and degassed prior to use. The column temperature was maintained at 25 °C, and the flow rate was 0.2 mL/min. Samples were centrifuged at 17,000 × g for 10 minutes before injection, and the injection volume was adjusted based on protein concentration (5–50 μg load). Elution was monitored at 280 nm for antibody samples, and data were analyzed by peak area normalization. The percentage of monomer, aggregates, and fragments was calculated based on the integrated peak areas.

#### Differential scanning calorimetry (DSC)

Thermal stability of the antibody was assessed using differential scanning calorimetry (DSC) on a VP-Capillary DSC system. The antibody was dialyzed into the respective formulation buffers and diluted to a final concentration of approximately 1 g/L. Samples and reference buffer were degassed and loaded into the sample and reference cells, respectively. The scan rate was set at 80 °C/h, with a temperature range of 10 °C to 100 °C. A minimum pressure of 35 PSI was maintained throughout the scan to prevent bubble formation. Buffer-buffer baseline scans were performed at least four times to ensure baseline stability. Following baseline subtraction, data were analyzed using the DSC analysis software, and the onset temperature of thermal unfolding (Tm Onset) was determined.

#### ALP activity assay

After culturing human bone marrow mesenchymal stem cells (hMSCs) under different conditions for 7 days, they were washed with ice bath, lysed at 4 °C and centrifuged. The supernatant was collected. The ALP activity was determined by using a commercial kit at 37 °C with the pNPP substrate for 5 minutes. The absorbance was read at 405 nm. A p-nitrophenol standard curve (2–20 nmol/well) was used. The ALP specific activity was calculated as nmol pNPP/min/mg protein (mU/mg), and the protein content was determined by the BCA method. Three replicates were set for each group, and the experiment was repeated three times.

#### Alizarin Red S staining and quantification

After 21 days of osteogenic induction, hMSCs were washed with PBS and fixed with 10% formaldehyde for 15 min at room temperature. The fixed cells were air-dried and washed three times with distilled water (5–10 min each wash). Alizarin Red S solution (1 mL/well) was added and incubated at room temperature for at least 20 min. After removal of the staining solution, the wells were washed four times with distilled water to eliminate unbound dye. Calcium deposits were visualized as orange-red nodules under light microscopy.

For quantitative analysis, images were captured under consistent illumination and magnification and saved as TIFF files. Using ImageJ software (NIH, USA), the images were converted to 8-bit grayscale, and the circular region of interest (ROI) corresponding to the microscopic field was selected to exclude background areas. A uniform threshold, determined based on the negative control (undifferentiated cells), was applied to all images to identify Alizarin Red S-positive areas. The percentage of positive area (% Area) was calculated for each well.

#### mRNA relative expression analysis

hMSCs (day 11 of osteogenic induction) and hPBMCs (day 7 of Transwell co-culture) were collected. Total RNA was extracted with TRIzol, reverse-transcribed to cDNA, and qPCR was performed using SYBR Green Master Mix. RPLP0 (28) and PPIA (29) served as reference genes for hMSCs and hPBMCs, respectively. The mRNA expression levels of ALP, Axin2, BGLAP, COL1A1, RUNX2, and SP7 were examined in hMSCs, while those of ACP5, CALCR, CTSK, ITGB3, MMP9, and NFATC1 were examined in hPBMCs. Relative expression was calculated by the 2^⁻ΔΔCt^ method, with the control group set to 100%. Primer sequences are listed in the **Supplementary Table 1**.

#### Sandwich ELISA for OPG and sRANKL concentrations

OPG and sRANKL concentrations were measured using commercial ELISA kits according to the manufacturer’s instructions. Briefly, samples or standards (100 μL/well) were added to capture antibody-coated wells and incubated at 37 °C for 90 min. After washing, biotinylated detection antibody was added and incubated for 60 min at 37 °C, followed by HRP conjugate for 30 min. After a final wash, TMB substrate was added and developed in the dark at 37 °C for approximately 15 min. The reaction was stopped, and the absorbance was read at 450 nm. Concentrations were calculated using a four-parameter logistic standard curve.

#### Tartrate-resistant acid phosphatase (TRAP) staining

After 7 days of Transwell co-culture, the culture medium was removed, and the cells in the upper chamber were fixed with 4% paraformaldehyde for 15–30 min, followed by three washes with distilled water. The cells were then permeabilized with 0.2% Triton X-100 for 20–30 min and washed three times with distilled water. TRAP working solution was added to cover the cells and incubated at 37 °C in the dark for 15–20 min. After three washes with distilled water, the nuclei were counterstained with hematoxylin. The cells were then dehydrated with absolute ethanol, air-dried, and examined under a light microscope. TRAP-positive multinucleated cells (containing three or more nuclei) with red/purple cytoplasmic staining were identified as mature osteoclasts and were indicated by red arrows in the representative images.

#### Statistical analysis

All experiments were independently repeated at least three times, and data were presented as mean ± standard deviation (SD). Statistical analysis and graph generation were performed using GraphPad Prism 10 software. Comparisons between two groups were made using an unpaired two-tailed Student’s t-test; comparisons among multiple groups were performed by one-way ANOVA followed by Tukey’s or Dunnett’s multiple comparisons test. A P value < 0.05 was considered statistically significant.

## Results

### Humanization and characterization of the anti-DKK-1 antibody

**Figure 2:**
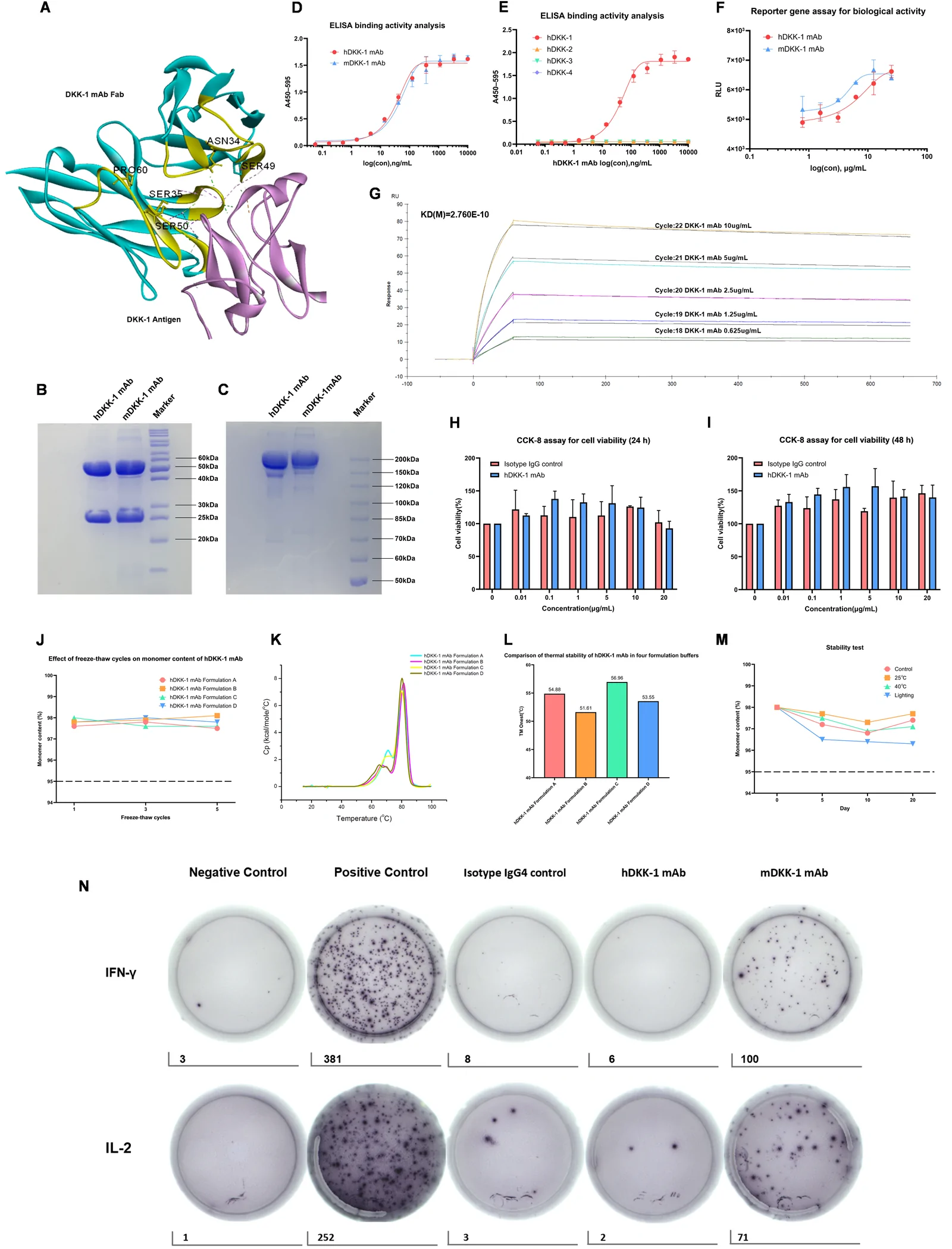
Humanization and Characterization of the Anti-DKK-1 Antibody. **(A)**, Schematic representation of the humanized anti-DKK-1 antibody. The back-mutation sites (A34N, Y49S, H35S, V50S, and A60P) introduced into the variable region and the predicted interaction model with the DKK-1 antigen are shown. **(B, C)**, SDS-PAGE analysis of the purified antibodies. Reducing **(B)** and non-reducing **(C)** SDS-PAGE of the humanized anti-DKK-1 antibody (hDKK-1 mAb) and the murine parental antibody (mDKK-1 mAb). **(D)**, Binding activity of hDKK-1 mAb and mDKK-1 mAb to immobilized DKK-1 determined by indirect ELISA. Nonlinear regression analysis using a four-parameter logistic model revealed an EC_50_ of 32.8 ng/mL for hDKK-1 mAb and 44.8 ng/mL for mDKK-1 mAb. **(E)**, Specificity of hDKK-1 mAb assessed by indirect ELISA against recombinant human DKK-1, DKK-2, DKK-3, and DKK-4. The antibody exhibited concentration-dependent binding to DKK-1 with an EC_50_ of 33.57 ng/mL, but showed no detectable cross-reactivity with DKK-2, DKK-3, or DKK-4. **(F)**, Functional activity assessed by the HEK-293/TCF-LEF Luc2p/Hygro reporter gene assay. Wnt3a-induced luciferase activity was suppressed by DKK-1, and this inhibition was reversed by hDKK-1 mAb and mDKK-1 mAb in a concentration-dependent manner. **(G)**, Surface plasmon resonance (SPR) sensorgrams for the binding of hDKK-1 mAb to immobilized DKK-1. The calculated equilibrium dissociation constant (KD) was 2.760 × 10⁻^10^ mol/L. **(H, I)**, CCK-8 assay for cell viability. Saos-2 cells were treated with various concentrations of hDKK-1 mAb or isotype IgG control for 24 h **(H)** and 48 h **(I)**. The antibody promoted cell proliferation in a time- and dose-dependent manner without overt cytotoxicity. **(J)**, Freeze-thaw stability of hDKK-1 mAb in four formulation buffers (Formulations A–D). Monomer content was determined by SEC after 1, 3, and 5 freeze-thaw cycles. All formulations maintained monomer content above 97% after 5 cycles. **(K, L)**, Thermal stability of hDKK-1 mAb in four formulation buffers assessed by differential scanning calorimetry (DSC). Formulation C exhibited the highest Tm onset (56.96 °C), approximately 5 °C higher than Formulation B (51.61 °C), indicating superior thermal stability. **(M)**, Stability of hDKK-1 mAb in Formulation C under various storage conditions (2–8 °C, 25 °C, 40 °C, and light exposure) monitored by SEC over 20 days. Monomer content remained stable under all conditions, with slight reduction under light exposure but remaining above 95%. **(N)**, ELISPOT assessment of cellular immunogenicity. PBMCs from healthy donors were stimulated with hDKK‑1 mAb, mDKK‑1 mAb, or human IgG4 isotype control. Anti‑CD3 antibody served as the positive control, and unstimulated cells as the negative control. IFN‑γ and IL‑2 spot‑forming cells (SFC) were counted per 1.5 × 10⁵ PBMCs. hDKK‑1 mAb did not elicit a significant T‑cell response compared with the isotype control, whereas mDKK‑1 mAb induced a robust response. Data are presented as mean ± SD (n = 3). Statistical significance was determined by one‑way ANOVA followed by Tukey's post‑hoc test. *P < 0.05, **P < 0.01, ***P < 0.001 vs. Control; ns, not significant.

The variable region sequences of the light and heavy chains of a mouse anti-DKK-1 antibody were obtained by 5′-RACE. Humanization of the mouse Fv region was achieved through CDR grafting combined with structure-guided rational back-mutation strategy. A homology model of the variable region was built using an antibody structure (PDB: 2XKN_AB) sharing 92.8% sequence identity with the mouse Fv as a template. Subsequently, the humanized antibody model was rigidly docked with DKK-1 antigen (PDB: 3S2K) using ZDOCK, followed by flexible optimization with RDOCK to determine the optimal binding conformation and evaluate key residue interactions. Based on these analyses, the humanized light and heavy chain variable region sequences were finalized, and the following back-mutations were introduced: A34N, Y49S, H35S, V50S, and A60P (**Figure 2A**). The constant region was selected as the IgG4 subtype.

The antibody gene was synthesized, cloned into an expression vector, transiently expressed in Expi293 cells, and purified by Protein A affinity chromatography. The purified product was analyzed by reducing and non-reducing SDS-PAGE, and compared with the murine anti-DKK-1 antibody as a control to confirm the correct expression of the antibody (**Figures 2B, C**).

The binding affinity of the humanized and murine anti-DKK-1 antibodies to DKK-1 was evaluated by indirect ELISA (**Figure 2D**). Nonlinear regression analysis using a four-parameter logistic model revealed an EC_50_ of 32.8 ng/mL (95% CI: 26.5–40.6 ng/mL) for the humanized antibody, which was slightly lower than that of the murine parental antibody (44.8 ng/mL, 95% CI: 37.8–53.1 ng/mL). Both antibodies displayed typical sigmoidal dose-response curves. These results indicate that humanization did not compromise the antigen-binding affinity of the antibody.

To further evaluate the specificity of the humanized anti-DKK-1 antibody, indirect ELISA was performed using recombinant human DKK-1, DKK-2, DKK-3, and DKK-4 as coated antigens. The humanized antibody bound to immobilized DKK-1 in a concentration-dependent manner, exhibiting a typical sigmoidal dose-response curve with an EC_50_ value of 33.57 ng/mL (**Figure 2E**). In contrast, no significant binding to DKK-2, DKK-3, or DKK-4 was observed across the entire concentration range tested, confirming that the humanized antibody specifically recognizes DKK-1 without cross-reactivity to other members of the Dickkopf family.

The biological activity of the antibody was assessed using a HEK-293/TCF-LEF Luc2p/Hygro reporter gene system. Wnt3a-induced luciferase activity was suppressed by DKK-1, and the ability of the humanized or murine anti-DKK-1 antibodies to reverse this inhibition was examined. As shown in **Figure 2F**, the inhibition was effectively reversed in a concentration-dependent manner by both antibodies, confirming the functional activity of the humanized antibody. Based on these results, 10 μg/mL was identified as the saturating concentration that fully relieved DKK-1-mediated Wnt suppression and was therefore selected for subsequent mechanistic studies.

Surface plasmon resonance (SPR) analysis yielded an equilibrium dissociation constant (KD) of 2.760 × 10⁻^10^ mol/L for the humanized antibody binding to DKK-1(**Figure 2G**).

To assess whether the humanized anti-DKK-1 antibody exerts any cytotoxic effects that may interfere with cell growth, we evaluated its impact on cell proliferation and viability using the human osteosarcoma cell line Saos-2, which is characterized by a short cell cycle and rapid proliferation, retains an intact Wnt/β-catenin signaling pathway, and exhibits sustained endogenous DKK-1-mediated pathway inhibition, making it an ideal model for evaluating anti-DKK-1 antibody function. The potential cytotoxicity of the humanized antibody was examined using the CCK-8 assay across a concentration range of 0.1 to 20 μg/mL. Compared with the isotype IgG control, the antibody did not exhibit overt cytotoxicity; at lower concentrations (0.1–10 μg/mL), cell viability was comparable to or slightly higher than that of the control group, with no signs of growth inhibition. Following 24 h of treatment, relative viabilities at 0.1 μg/mL and 1 μg/mL were 137.6% and 132.5%, respectively, versus 112.6% and 110.2% for the isotype control (**Figure 2H**). After 48 h, viabilities reached 155.8% at 1 μg/mL and 156.9% at 5 μg/mL (isotype control: 137.1% and 119.0%, respectively) (**Figure 2I**). Even at the highest concentration tested (20 μg/mL), the antibody did not reduce cell viability below control levels, although the proliferative effect was attenuated, suggesting a possible plateau or mild non-specific cytotoxicity only at supra-therapeutic concentrations. Collectively, these results demonstrate that the humanized anti-DKK-1 antibody exhibits no significant cytotoxicity within the tested concentration range.

To identify an optimal formulation for the humanized anti-DKK-1 antibody, the antibody was buffer-exchanged into four formulation buffers (Formulations A–D) and evaluated for freeze-thaw stability and thermal stability. To assess the physical stability of the antibody under stress conditions, each formulation was subjected to 1, 3, and 5 freeze-thaw cycles, and monomer content was monitored by size-exclusion chromatography (SEC). As shown in **Figure 2J** (**Supplementary Table 2**), all four formulations maintained monomer content above 97% after 5 freeze-thaw cycles, with no significant increase in aggregates or fragments under any of the tested conditions. Notably, Formulation C consistently exhibited stable monomer content (97.6–98.0%) throughout the freeze-thaw cycles. To further evaluate the conformational stability of the antibody in each formulation, differential scanning calorimetry (DSC) was performed. As shown in **Figures 2K, L**, Formulation C exhibited the highest Tm onset (56.96 °C) among the four tested formulations, approximately 5 °C higher than Formulation B (51.61 °C), indicating superior thermal stability. Based on these results, Formulation C was selected as the optimal buffer system for subsequent stability studies due to its consistent freeze-thaw stability profile and superior thermal stability.

The antibody formulated in Buffer C was then subjected to stability testing under various storage conditions, including 2–8 °C (Control), 25 °C, 40 °C, and light exposure. SEC analysis revealed that the monomer content remained stable under all temperature conditions, with no significant degradation observed (**Figure 2M**). Under light exposure, a slight decrease in monomer content was noted, but it remained above 95%, confirming the favorable stability profile of the antibody in Formulation C.

To evaluate the cellular immunogenicity of the humanized anti-DKK-1 antibody, IFN-γ and IL-2 ELISPOT assays were performed using peripheral blood mononuclear cells (PBMCs) from healthy donors. As shown in **Figure 2N**, the positive control (anti-CD3 antibody) induced robust T-cell responses, with 381 spot-forming cells (SFC) per 1.5 × 10^5^ PBMCs for IFN-γ and 252 SFC for IL-2, confirming the functional integrity of the assay system. The negative control (unstimulated cells) exhibited minimal background spots (IFN-γ: 3 SFC; IL-2: 1 SFC). The human IgG4 isotype control also showed low spot counts (IFN-γ: 8 SFC; IL-2: 3 SFC), indicating that the IgG4 backbone itself did not elicit a significant T-cell response. Notably, the humanized anti-DKK-1 antibody (hDKK-1 mAb) did not induce a significant increase in either IFN-γ (6 SFC) or IL-2 (2 SFC) spots compared with the isotype control or negative control. In contrast, the murine anti-DKK-1 antibody (mDKK-1 mAb) elicited a markedly higher T-cell response (IFN-γ: 100 SFC; IL-2: 71 SFC), confirming the immunogenicity of the parental murine antibody. These results demonstrate that the humanization strategy successfully reduced the T-cell immunogenicity potential of the antibody. The data suggest that the humanized anti-DKK-1 antibody has a low risk of inducing cellular immune responses, supporting its favorable safety profile for further development.

### Wnt/β-Catenin signaling governs early osteogenic differentiation and the OPG/RANKL axis in hMSCs

**Figure 3:**
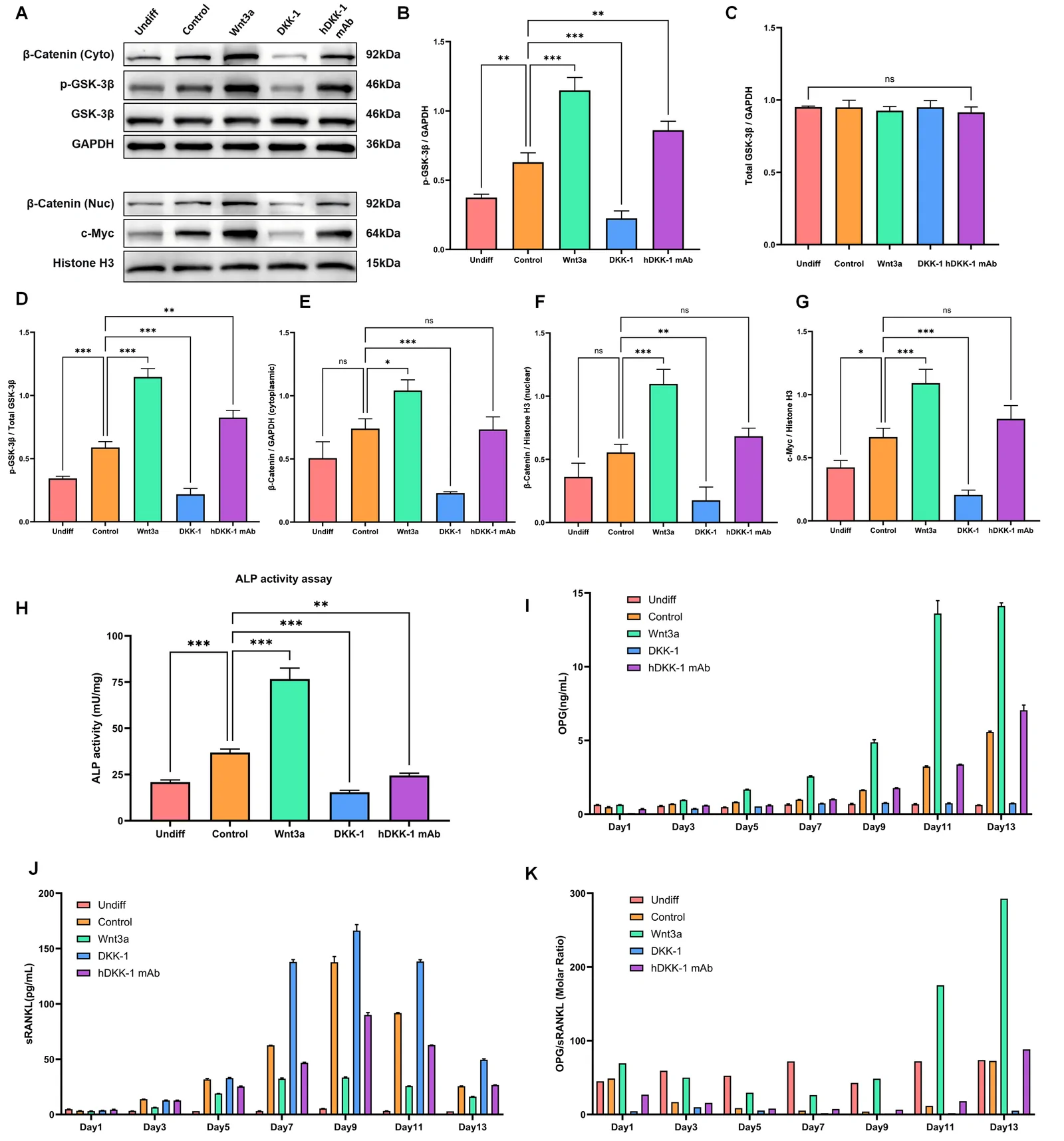
Wnt/β-catenin signaling activation, osteogenic differentiation, and OPG/sRANKL regulation by the humanized anti-DKK-1 antibody. **(A)**, hMSCs were cultured under different conditions for 7 days, followed by subcellular fractionation to separate cytoplasmic and nuclear proteins for Western blot analysis. GAPDH and Histone H3 served as loading controls for cytoplasmic and nuclear fractions, respectively. **(B–G)**, Western blot analysis of Wnt/β-catenin signaling components. **(B)**, Ratio of p-GSK-3β (Ser9) to GAPDH. **(C)**, Ratio of total GSK-3β to GAPDH. **(D)**, Ratio of p-GSK-3β (Ser9) to total GSK-3β, reflecting GSK-3β inactivation. **(E)**, Ratio of cytoplasmic β-catenin to GAPDH. **(F)**, Ratio of nuclear β-catenin to Histone H3. **(G)**, Ratio of nuclear c-Myc to Histone H3. Data are presented as mean ± SD (n = 3). *P < 0.05, **P < 0.01, ***P < 0.001 vs. Control; ns, not significant. **(H)**, Alkaline phosphatase (ALP) activity of hMSCs on day 7 of osteogenic induction. Cells were cultured under five conditions: undifferentiated (Undiff), osteogenic induction medium (Control), Wnt3a (50 ng/mL), DKK-1 (200 ng/mL), and hDKK-1 mAb (10 µg/mL + 200 ng/mL DKK-1). ALP activity was measured using a p_nitrophenyl phosphate (pNPP) substrate and normalized to total protein content. **(I)**, Time course of OPG concentrations in culture supernatants measured by ELISA (days 1–13). **(J)**, Time course of sRANKL concentrations in culture supernatants measured by ELISA (days 1–13). **(K)**, OPG/sRANKL molar ratio calculated using molecular weights of 60 kDa for OPG and 20 kDa for sRANKL. Data are presented as mean ± SD (n = 3). Statistical significance was determined by one-way ANOVA followed by Tukey’s post-hoc test. *P < 0.05, **P < 0.01, ***P < 0.001 vs. Control; ns, not significant.

To investigate whether the humanized anti-DKK-1 antibody reactivates Wnt/β-catenin signaling, hMSCs were cultured for 7 days under various conditions, followed by subcellular fractionation and Western blot analysis. Cytoplasmic fractions were probed for total GSK-3β, total β-catenin, and p-GSK-3β (Ser9) with GAPDH as the loading control, while nuclear fractions were probed for β-catenin and c-Myc with Histone H3 as the loading control (**Figure 3A**). At the upstream level, the ratio of p-GSK-3β (Ser9) to total GSK-3β, which reflects GSK-3β inactivation, was markedly reduced by DKK-1 treatment (0.22 vs. 0.59 in Control, P < 0.001), indicating sustained GSK-3β activity and consequent β-catenin degradation; notably, this ratio was restored to 0.82 by the anti-DKK-1 antibody, exceeding the Control level and confirming effective reversal of Wnt suppression (**Figures 3B–D**). Accordingly, in the midstream step, DKK-1 drastically decreased cytoplasmic β-catenin (0.23 vs. 0.74 in Control), whereas the antibody restored it to 0.73, comparable to Control. More importantly, nuclear β-catenin accumulation—the hallmark of canonical Wnt pathway activation, (**Figures 3E, F**)—was profoundly reduced by DKK-1 (0.17 vs. 0.55 in Control, P < 0.001), and was restored to 0.68 by the antibody, a level not significantly different from Control (P = 0.49). As a positive control, Wnt3a induced the highest nuclear β-catenin level (1.10). Downstream of this cascade, c-Myc, a classical Wnt/β-catenin target gene, was strongly suppressed by DKK-1 (0.21 vs. 0.66 in Control, P < 0.001), and its expression recovered to 0.81 upon antibody treatment (P < 0.001 vs. DKK-1; P = 0.18 vs. Control, **Figure 3G**), confirming transcriptional activation following DKK-1 neutralization. Collectively, the expression levels of all examined proteins consistently followed the gradient: DKK-1 < Undiff < Control ≤ hDKK-1 mAb < Wnt3a, demonstrating that the humanized anti-DKK-1 antibody effectively reinstates Wnt/β-catenin signaling by promoting β-catenin nuclear translocation, inactivating GSK-3β, and activating downstream target gene transcription.

To evaluate the early osteogenic differentiation capacity of hMSCs under different conditions, ALP activity was measured on day 7 of culture. ALP is a well-established early marker of osteoblastic differentiation, and its activity positively correlates with subsequent matrix mineralization. ALP activity was calculated using a concurrently generated p-nitrophenol standard curve (linear regression: Y = 9.3906X + 0.0498, R² = 0.9979) and expressed as specific activity per milligram of protein (mU/mg protein). As shown in the figure (**Figure 3H**), ALP activity in the osteogenic induction control group was 34.88 ± 3.5 mU/mg, approximately 1.7-fold higher than that of the undifferentiated control (P < 0.05). This increase was consistent with numerous reports demonstrating that standard osteogenic induction medium (containing ascorbic acid, β-glycerophosphate, and dexamethasone) significantly elevates ALP activity in hMSCs within 7 days. Treatment with recombinant Wnt3a further increased ALP activity to 79.80 ± 8.0 mU/mg, 2.3-fold higher than the control, in line with previous findings showing that Wnt3a enhanced BMP9-induced ALP activity and mineralization in mesenchymal stem cells. In contrast, treatment with DKK-1 significantly reduced ALP activity to 14.84 ± 1.5 mU/mg, even lower than the undifferentiated control, confirming that DKK-1 is a potent inhibitor of canonical Wnt signaling and effectively blocked dexamethasone-induced osteogenic differentiation. The residual ALP activity in the DKK-1 group may be attributable to BMP signaling, which was also triggered by dexamethasone and was unaffected by DKK-1. Importantly, co-treatment with the anti-DKK-1 monoclonal antibody partially restored ALP activity to 26.04 ± 2.6 mU/mg, corresponding to approximately 75% of the control level and representing a significant recovery compared with DKK-1 alone. This restoration was consistent with our earlier observation that the antibody rescued OPG secretion and further supported the functional neutralization of DKK-1. Collectively, these data demonstrated that Wnt/β-catenin signaling promoted early osteogenic differentiation of hMSCs, and that the anti-DKK-1 antibody effectively counteracted exogenous DKK-1 inhibition, restoring ALP activity toward normal levels.

OPG is regulated by Wnt/β-catenin signaling. Undifferentiated hMSCs secrete only minimal OPG, maintaining a high RANKL/OPG ratio that can initiate and support osteoclastogenesis, whereas mature osteoblasts exhibit a greatly reduced RANKL/OPG ratio and lose this supportive capacity. In order to determine the optimal time point for initiating co-culture, we need to identify the peak time point at which human bone marrow mesenchymal stem cells secrete OPG. To this end, we cultured hMSCs in different media and quantified OPG concentrations in culture supernatants by sandwich ELISA (**Figure 3I**). We found that undifferentiated hMSCs secreted negligible OPG, while osteogenic induction conferred OPG secretory ability. Wnt/β-catenin signaling was identified as a key regulatory pathway of OPG expression: Wnt3a robustly promoted OPG secretion, while DKK-1 completely suppressed it. The humanized anti-DKK-1 antibody effectively reversed DKK-1-mediated inhibition and restored OPG secretion to levels approaching or even exceeding the induction control. Intergroup differences in OPG secretion were most pronounced on day 11 of culture, making it a suitable starting point for subsequent co-culture. In osteoblast–osteoclast co-culture, selecting the appropriate initiation time is crucial. Starting too early means osteoblasts are insufficiently differentiated and OPG secretion is inadequate; starting too late may allow cells to enter the mineralization phase with altered OPG/RANKL ratios. In the present co-culture model, the dominant form of RANKL is soluble RANKL (sRANKL). Osteoblast-derived RANKL is primarily membrane-bound, and soluble RANKL is secreted at the pg/mL level; therefore, exogenous sRANKL (30 ng/mL) must be supplemented to induce osteoclast differentiation. Based on molar ratio conversion, OPG secreted by the higher-density hMSCs in the lower chamber of the 24-well Transwell system (at least 5–10 times the number of upper-chamber hPBMCs, as commonly reported) is sufficient to completely neutralize the added exogenous RANKL, thereby achieving the intended experimental design.

To evaluate the influence of different treatments on sRANKL secretion by osteoblasts, we collected culture supernatants on days 1, 3, 5, 7, 9, 11, and 13 and measured sRANKL levels by ELISA. As shown in the figure (**Figure 3J**), sRANKL concentrations in the undifferentiated group remained consistently low (3–5 pg/mL), indicating negligible secretion by undifferentiated hMSCs. The osteogenic induction control group displayed a unimodal pattern: sRANKL began to increase on day 3 (13.9 pg/mL), peaked on day 9 (137.7 pg/mL), and then declined to 25.6 pg/mL by day 13, consistent with the transient RANKL upregulation during mid-stage osteogenic differentiation. The Wnt3a-stimulated group showed a markedly lower sRANKL peak (~32 pg/mL on days 5–7) that occurred earlier and then gradually declined, suggesting that Wnt3a accelerates osteogenic differentiation and suppresses RANKL expression. The DKK-1 inhibition group maintained persistently high sRANKL levels, reaching a maximum of 166.3 pg/mL on day 9 and remaining at 138.3 pg/mL on day 11, indicating that Wnt signal blockade by DKK-1 arrests osteoblasts in a pro-osteoclastogenic state with enhanced RANKL secretion. The DKK-1 antibody rescue group exhibited intermediate sRANKL levels, with a peak of 90.0 pg/mL on day 9 and a decline to 62.7 pg/mL on day 11, confirming that the antibody effectively reverses the DKK-1 effect.

OPG and sRANKL concentrations determined by ELISA were expressed in pg/mL. Mass concentrations were converted to molar concentrations using molecular weights of 60 kDa for OPG and 20 kDa for sRANKL, and the OPG/sRANKL molar ratio was calculated (**Figure 3K**). In the osteogenic control group, the molar ratio decreased sharply from day 1 to day 9 (from 48.8 to 3.99) and then rose to 72.74 on day 13, reflecting the typical transition from a pro-osteoclastogenic to an anti-osteoclastogenic state during osteoblast differentiation. DKK-1 treatment maintained the ratio at an extremely low level (≤9.8 throughout the culture period), indicating persistent suppression of osteoblast-mediated anti-osteoclastogenic capacity. In contrast, Wnt3a treatment elevated the ratio to 292.71 on day 13, demonstrating substantially enhanced OPG dominance. Importantly, co-treatment with the anti-DKK-1 antibody restored the ratio to 88.33 on day 13, which was even slightly higher than the control value of 72.74, confirming effective reversal of the DKK-1-induced functional defect in osteoblasts.

Based on these dynamic profiles, day 11 of osteogenic induction was chosen as the initiation point for the osteoblast–osteoclast Transwell co-culture. At this time point, sRANKL concentrations differed significantly among groups: undifferentiated 3.2 pg/mL, induction control 91.7 pg/mL, Wnt3a 25.8 pg/mL, DKK-1 138.3 pg/mL, and DKK-1 antibody 62.7 pg/mL. Notably, these endogenous sRANKL levels are far below the concentrations required for efficient *in vitro* osteoclastogenesis. Therefore, during co-culture, exogenous recombinant human sRANKL was uniformly supplemented at 30 ng/mL (30,000 pg/mL) in all groups to provide a consistent osteoclastogenic induction background. This design allowed independent assessment of the ability of osteoblast-derived OPG to neutralize exogenous RANKL, without interference from variations in endogenous sRANKL.

### Osteogenic gene expression and mineralization in hMSCs.

**Figure 4:**
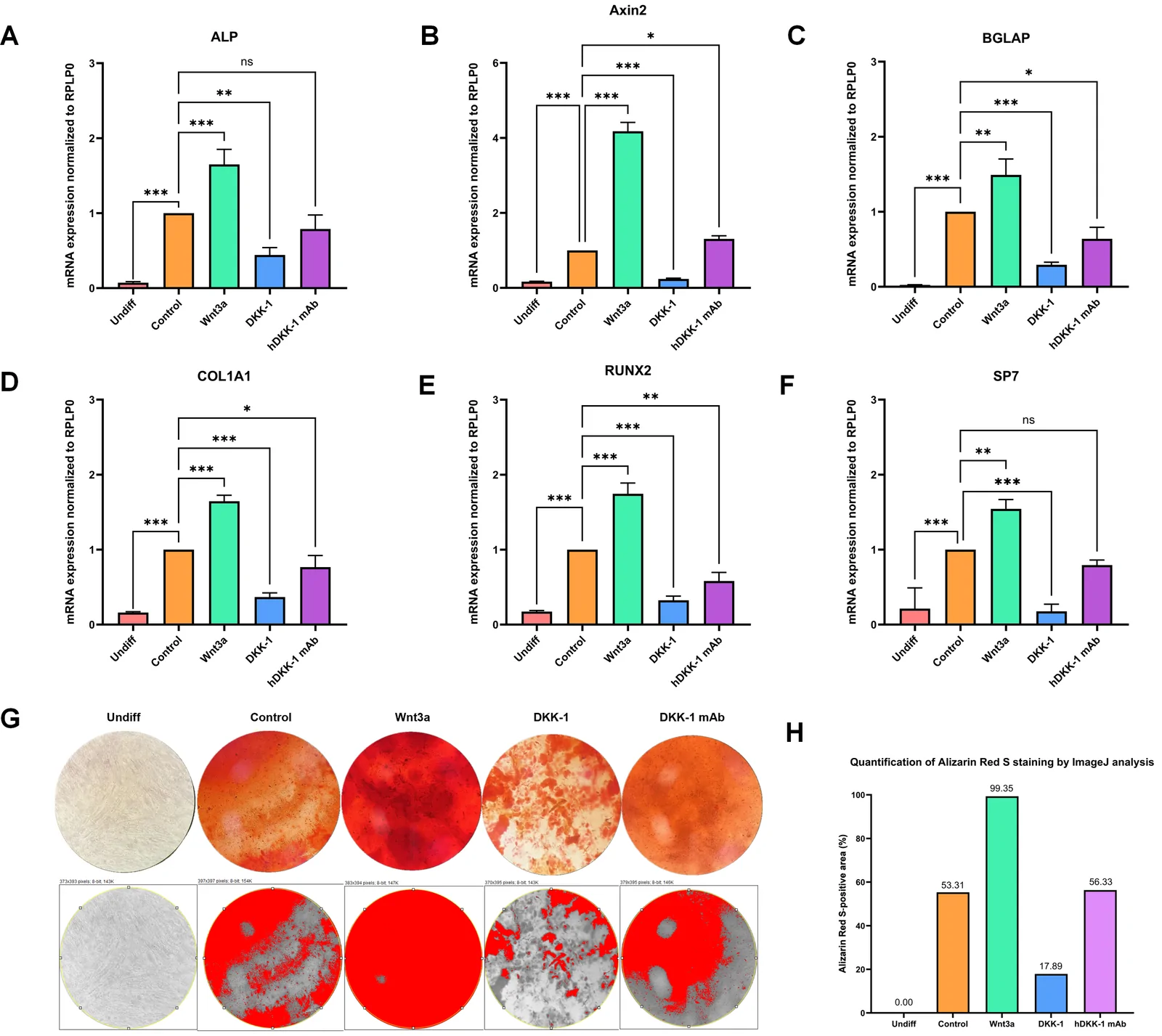
Osteogenic gene expression and mineralization in hMSCs. **(A–F)**, Relative mRNA expression of osteogenic marker genes in hMSCs on day 11 of osteogenic induction. Expression levels of **(A)** ALP, **(B)** Axin2, **(C)** BGLAP, **(D)** COL1A1, **(E)** RUNX2, and **(F)** SP7 were normalized to RPLP0 and expressed as fold change relative to the Control group (Control = 1). Data are presented as mean ± SD (n = 3). *P < 0.05, **P < 0.01, ***P < 0.001 vs. Control; ns, not significant. **(G)**, Quantitative analysis of Alizarin Red S staining by ImageJ. The percentage of Alizarin Red S-positive area (% Area) was calculated for each group. **(H)**, Representative images of Alizarin Red S staining of mineralized nodules on day 21. Statistical significance was determined by one-way ANOVA followed by Tukey’s post-hoc test. *P < 0.05, **P < 0.01, ***P < 0.001 vs. Control; ns, not significant.

After 14 days of culture under osteogenic induction, the mRNA expression of osteogenic differentiation genes was analyzed, the undifferentiated group showed consistently low expression levels across all genes examined (**Figures 4A–F**) (**Supplementary Table 3**). DKK-1 treatment significantly suppressed the expression of osteogenic genes compared with the Control group, although the expression levels remained higher than those in the undifferentiated group, likely due to the residual osteogenic drive of dexamethasone via non-canonical pathways. In contrast, Wnt3a stimulation robustly upregulated all osteogenic genes, consistent with its role as a potent activator of canonical Wnt signaling. The anti-DKK-1 antibody partially restored the expression of these genes, with recovery levels approaching those of the Control group. Notably, Axin2, a direct transcriptional target of the Wnt/β-catenin pathway, was significantly upregulated in the hDKK-1 mAb group compared with the Control group (1.31-fold, P = 0.0474) (**Figure 4B**), confirming effective restoration of Wnt signaling activity upon DKK-1 neutralization. Collectively, these results demonstrate that the humanized anti-DKK-1 antibody effectively reinstates Wnt/β-catenin signaling and promotes the transcription of osteogenic genes in hMSCs.

After 21 days of osteogenic induction, the mineralization capacity of hMSCs under different conditions was assessed by Alizarin Red S staining (**Figures 4G, H**). As expected, the undifferentiated control group (Undiff), cultured in basal medium without osteogenic supplements, showed no mineralized nodules, confirming the absence of spontaneous osteogenic differentiation. In contrast, the osteogenic control group (Control), cultured in osteogenic induction medium containing ascorbic acid, β-glycerophosphate, and dexamethasone, displayed distinct mineralized nodules, indicating successful osteoblast differentiation and matrix mineralization. Treatment with Wnt3a markedly enhanced mineralization, resulting in extensive and densely stained calcium deposits compared with the Control group, consistent with the well-established pro-osteogenic role of Wnt/β-catenin signaling. Conversely, exposure to DKK-1 significantly reduced the formation of mineralized nodules relative to the Control group, although a small number of nodules remained, likely attributable to dexamethasone-induced non-canonical pathways (e.g., BMP signaling) that are not blocked by DKK-1. Importantly, co-treatment with the anti-DKK-1 monoclonal antibody effectively rescued the mineralization defect caused by DKK-1, restoring the number and intensity of Alizarin Red S-positive nodules to a level comparable to that of the Control group. Quantitative analysis using ImageJ software revealed that the Alizarin Red S-positive area was 0.00% in the Undiff group, 53.31% in the Control group, 99.35% in the Wnt3a group, 17.89% in the DKK-1 group, and 56.33% in the hDKK-1 mAb group. These quantitative data corroborate the visual observations and confirm that the humanized anti-DKK-1 antibody specifically neutralizes DKK-1, reinstates Wnt/β-catenin signaling, and thereby restores the osteogenic differentiation and mineralization capacity of hMSCs.

### The humanized anti-DKK-1 antibody suppresses osteoclastogenic gene expression via OPG-mediated neutralization of RANKL in Transwell co-culture

After 7 days of osteogenesis-osteoclastogenesis Transwell co-culture, hPBMC-derived osteoclasts in the upper Transwell chamber were harvested, and the relative mRNA expression of six osteoclast marker genes—ACP5, CALCR, CSTK, ITGB3, MMP9, and NFATC1—was quantified by real-time PCR using PPIA as the reference gene. All groups uniformly received 30 ng/mL M-CSF and 30 ng/mL sRANKL; hence, differences in gene expression primarily reflect the OPG-mediated neutralization capacity of the underlying hMSCs.

**Figure 5:**
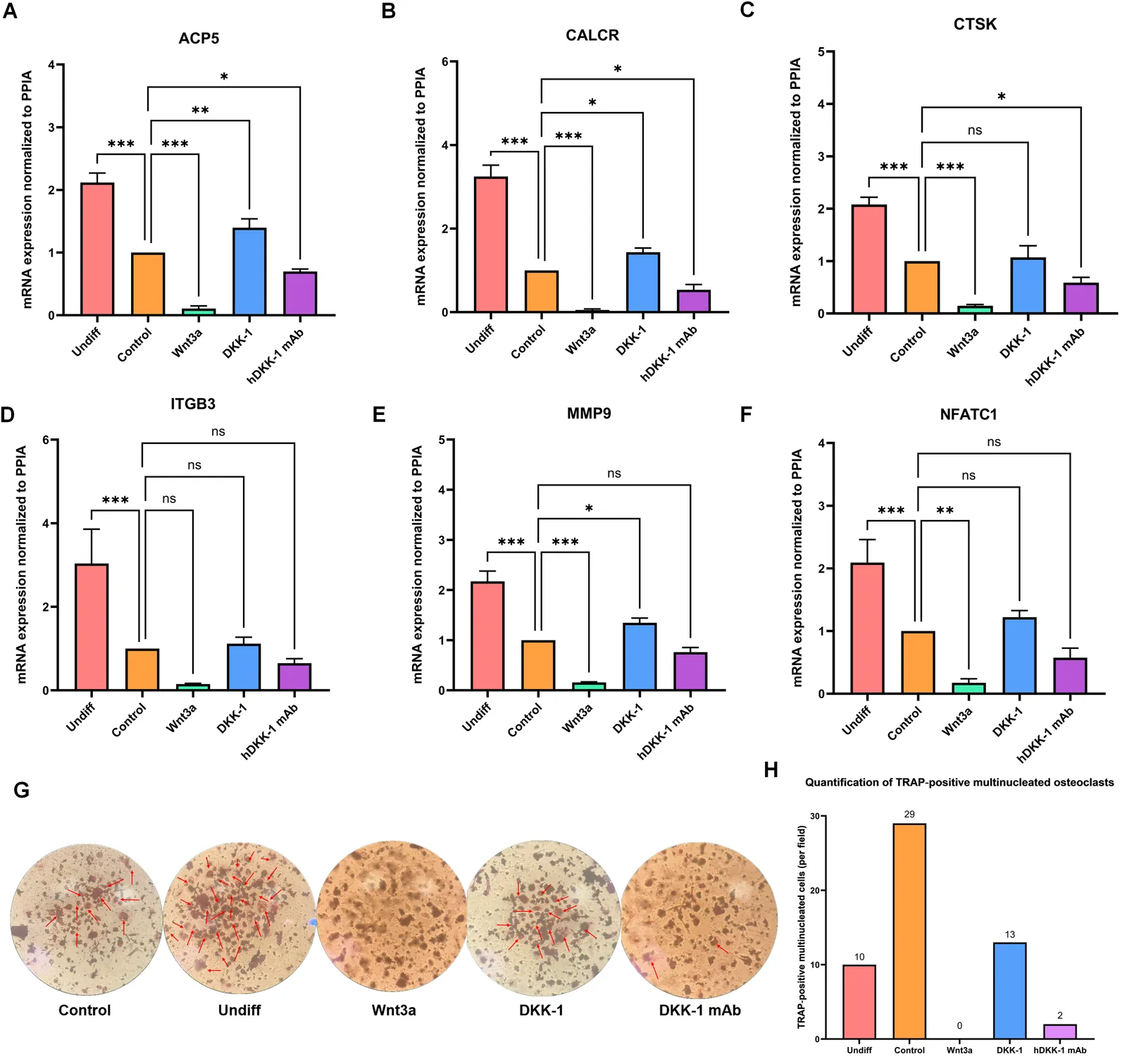
Humanized anti-DKK-1 antibody suppresses osteoclastogenic gene expression and osteoclast differentiation in Transwell co-culture. **(A–F)**, After 7 days of co-culture, hPBMCs in the upper chamber were collected, and the relative mRNA expression of osteoclast marker genes ACP5, CALCR, CSTK, ITGB3, MMP9, and NFATC1 was determined by RT-qPCR using PPIA as the reference gene (Control group = 1). All groups uniformly received exogenous M-CSF (30 ng/mL)and sRANKL (30 ng/mL). Data are presented as mean ± SD (n = 3). *P < 0.05, **P < 0.01, ***P < 0.001 vs. Control; ns, not significant. **(G)**, After 7 days of co-culture, hPBMCs in the upper chamber were stained for TRAP (tartrate-resistant acid phosphatase) and counterstained with hematoxylin. Representative micrographs (magnification × 40) show TRAP-positive multinucleated cells. Control: moderate OPG → scattered osteoclasts; Undiff: undifferentiated hMSCs (no OPG) → abundant TRAP-positive cells; Wnt3a: high OPG → extremely rare TRAP-positive cells, but mononuclear aggregates present (hematoxylin); DKK-1: low OPG → numerous TRAP-positive cells; hDKK-1 mAb: restored OPG → significantly fewer TRAP-positive cells than Control. **(H)**, Number of TRAP-positive multinucleated cells (≥3 nuclei) per field.

A highly consistent inter-group trend was observed across all six genes (**Figures 5A–F**). The Undiff group, in which undifferentiated hMSCs secrete negligible OPG, exhibited the highest expression levels, ranging from 1.94- to 3.88-fold relative to Control (ITGB3: 3.88; CALCR: 3.16; ACP5: 2.27), indicating that the exogenous sRANKL was largely unopposed and drove maximal osteoclast differentiation. In the Wnt3a group, osteoclast gene expression was profoundly suppressed (4.5–16.9% of Control; ACP5: 0.06; CALCR: 0.04; ITGB3: 0.13; NFATC1: 0.13), consistent with robust OPG secretion by Wnt3a-stimulated osteoblasts that efficiently neutralized exogenous sRANKL and nearly shut down the osteoclastogenic program. The DKK-1 group showed expression levels close to or moderately above Control (ACP5: 1.26; CALCR: 1.47; NFATC1: 1.22; MMP9: 1.26), whereas CSTK (0.83) and ITGB3 (0.94) were comparable to Control, consistent with the near-complete suppression of OPG by DKK-1 and the consequent lack of antagonism toward exogenous sRANKL. In the hDKK-1 mAb rescue group, all osteoclast genes were expressed at intermediate levels between the Control and Wnt3a groups (40–84% of Control; ACP5: 0.66; CALCR: 0.40; NFATC1: 0.43; CSTK: 0.47), reflecting partial restoration of OPG secretion and a corresponding degree of osteoclast inhibition. Notably, this gene-expression gradient closely mirrors the OPG protein levels measured in the osteoblast compartment, where the anti-DKK-1 antibody restored OPG to approximately 75% of the Control level. Together, these results provide genetic evidence from the osteoclast side that the Wnt/β-catenin/OPG axis quantitatively governs osteoclast differentiation in this co-culture system, and that the humanized anti-DKK-1 antibody effectively suppresses osteoclastogenic gene expression by rescuing OPG-mediated RANKL neutralization.

After 7 days of Transwell co-culture of hPBMCs (upper chamber) with hMSCs (lower chamber) in medium containing exogenous M-CSF and sRANKL, osteoclast differentiation was assessed by TRAP staining, with the number of TRAP-positive multinucleated cells (≥3 nuclei) per field quantified (**Figures 5G, H**). In the undifferentiated control group (Undiff), hMSCs secreted negligible OPG and failed to neutralize exogenous RANKL, resulting in abundant TRAP-positive multinucleated giant cells (29 ± 5 per field) that served as a reference for maximal osteoclastogenesis. In contrast, the osteogenic control group (Control) exhibited moderate OPG secretion that partially neutralized RANKL, significantly reducing osteoclast numbers to 10 ± 2 per field with only scattered osteoclasts observed. Wnt3a treatment markedly enhanced OPG production, efficiently neutralizing RANKL and nearly abolishing osteoclast differentiation (0 ± 1 per field), although hematoxylin staining revealed mononuclear aggregates, indicating that high OPG levels specifically prevented precursor fusion. Conversely, DKK-1 treatment abrogated Wnt/β-catenin signaling, drastically reducing OPG secretion and impairing RANKL neutralization, which promoted osteoclast differentiation to 13 ± 3 per field, significantly higher than Control and approaching Undiff levels. Importantly, co-treatment with the anti-DKK-1 antibody restored OPG secretion, partially neutralized RANKL, and markedly reduced osteoclast numbers to 2 ± 1 per field, significantly lower than Control and comparable to Wnt3a, confirming that the antibody effectively reversed DKK-1-induced osteoclastogenesis. Collectively, these quantitative TRAP data demonstrate that the humanized anti-DKK-1 antibody reinstates osteoblast-mediated suppression of osteoclastogenesis through restoration of Wnt/β-catenin signaling and upregulation of OPG secretion.

## Discussion

Our results align with the established role of OPG as a key inhibitor of osteoclastogenesis. OPG is a soluble decoy receptor for RANKL, predominantly produced by osteoblasts, and the RANKL/OPG ratio is a critical determinant of bone mass in normal and disease states. Wnt/β-catenin signaling has been shown to upregulate OPG transcription, thereby suppressing bone resorption. In our study, the OPG/sRANKL molar ratio exhibited a strong inverse correlation with osteoclast gene expression across all experimental groups: the Wnt3a group showed the highest ratio (292.71) and lowest osteoclast marker expression, while the DKK-1 group showed the lowest ratio (5.05) and highest osteoclast marker expression. The anti-DKK-1 antibody restored the ratio to a level comparable to the control, and this was faithfully reflected in suppressed osteoclast differentiation. Although we did not perform direct OPG loss-of-function experiments, the quantitative relationship between OPG availability and osteoclast differentiation, together with extensive literature support, provides compelling evidence for OPG’s central role in mediating the antibody’s anti-resorptive effects.

To put our approach into clinical context, **Table 1** compares currently approved osteoporosis therapies with our anti-DKK-1 strategy.

**Table 1:** Comparison of existing osteoporosis treatments with the anti-DKK-1 antibody approach (2, 3, 30–32).

Treatment class	Representative agents	Mechanism	Advantages	Limitations
Antiresorptive agents	Bisphosphonates (e.g., alendronate, zoledronic acid), Denosumab	Inhibit osteoclast activity, reduce bone resorption	Effectively reduce fracture risk; can be used long-term (bisphosphonates)	Do not actively rebuild lost bone; may excessively suppress bone turnover; long-term use associated with atypical femoral fractures and osteonecrosis of the jaw
Anabolic agents	Teriparatide, Abaloparatide	Stimulate osteoblast activity, promote new bone formation	Significantly increase bone mineral density and improve microarchitecture	Require daily subcutaneous injection; treatment duration limited to ≤2 years; may increase bone resorption; bone loss occurs rapidly after discontinuation
Dual-action agent (approved)	Romosozumab	Anti-sclerostin antibody; increases bone formation and decreases resorption via OPG upregulation	First dual-action agent; approved for high-risk osteoporosis	Cardiovascular safety concerns (increased risk of myocardial infarction); sclerostin is predominantly expressed by osteocytes, limiting the target distribution
Anti-DKK-1 antibody (our approach, investigational)	Humanized anti-DKK-1 mAb (IgG4)	Neutralizes DKK-1 → restores Wnt/β-catenin → upregulates OPG → suppresses RANKL-driven osteoclastogenesis	Dual anabolic and anti-resorptive through a single target; broader expression of DKK-1 (osteoblasts and osteocytes) may offer complementary benefits in conditions where DKK-1 is the dominant Wnt antagonist	Currently in preclinical/translational stage; compensatory upregulation of SOST may limit efficacy; further in vivo and clinical studies needed

In this study, we generated a humanized anti-DKK-1 monoclonal antibody and demonstrated its dual functionality through the Wnt/β-catenin/OPG axis. Our Transwell co-culture experiments showed that neutralizing DKK-1 relieves Wnt inhibition in osteoblasts, upregulates OPG secretion, and effectively neutralizes exogenous RANKL, thereby suppressing osteoclast differentiation. These findings mechanistically link DKK-1 blockade to both anabolic and anti-resorptive outcomes. While donor-to-donor variability is an inherent feature of primary human cell-based assays, the consistent relative trends observed across multiple independent donors in this study support the robustness of our conclusions.

Romosozumab, an anti-sclerostin antibody, has validated the clinical potential of targeting a Wnt inhibitor for osteoporosis (2, 33). However, sclerostin is predominantly expressed by osteocytes, whereas DKK-1 is more broadly expressed in osteoblasts and osteocytes and is upregulated under pathological conditions such as estrogen deficiency and glucocorticoid excess (34, 35). Our results add to the growing evidence that DKK-1 neutralization may offer complementary benefits, particularly in disease states where DKK-1 is the dominant antagonist. The observed increase in OPG secretion and the reciprocal suppression of sRANKL upon Wnt3a treatment are consistent with the established role of canonical Wnt signaling in promoting OPG transcription (10). Conversely, the sustained low OPG/sRANKL ratio in DKK-1-treated cells mimics the pro-osteoclastogenic state of undifferentiated hMSCs, in line with the concept that the osteoblast differentiation stage determines the RANKL/OPG balance (36, 37). Importantly, the anti-DKK-1 antibody restored the molar ratio to a level that strongly inhibited osteoclastogenesis, and this was faithfully reflected in the suppressed expression of osteoclast marker genes in co-cultured hPBMCs (38). The strong inverse correlation between OPG protein levels and osteoclast gene expression supports a quantitative model in which the degree of Wnt activation in osteoblasts proportionally determines the extent of osteoclast inhibition (39).

The choice of IgG4 constant region was deliberate. IgG4 exhibits low affinity for Fcγ receptors and complement, minimizing unwanted effector functions such as ADCC and CDC (40). Given that DKK-1 is a soluble antagonist, the primary therapeutic goal is ligand neutralization rather than depletion of DKK-1-expressing cells. An IgG4 backbone is thus appropriate for a chronic disease setting.

The Transwell co-culture system allows dissection of paracrine crosstalk but does not fully recapitulate the three-dimensional bone marrow niche, mechanical loading, or the contributions of osteocytes and immune cells (41–43). Moreover, because endogenous sRANKL secretion by hMSCs is at the pg/mL level, exogenous sRANKL was uniformly added to all groups to drive efficient osteoclastogenesis (25). This design intentionally isolated OPG-mediated regulation as the primary variable; however, it may have masked subtle modulations of endogenous RANKL production by the antibody. Future studies using more complex models, such as three-dimensional co-cultures or osteoblast-osteocyte-osteoclast tri-cultures, would be valuable.

Several factors may explain the incomplete rescue observed in some functional assays—approximately 75% recovery in ALP activity and 70% recovery in mineralization. One key factor is the compensatory upregulation of other endogenous Wnt antagonists, particularly sclerostin (SOST). Studies have demonstrated that DKK-1 inhibition can increase SOST expression, suggesting a potential compensatory mechanism that limits the anabolic response to DKK-1 blockade (32). Consistent with this, bispecific antibodies targeting both sclerostin and DKK-1 have been shown to produce superior bone formation compared with monospecific antibodies alone. This reciprocal regulation represents a major limitation of single-target strategies and may explain the partial rescue observed in our assays. Additionally, the residual ALP activity and mineralization in DKK-1-treated cells suggest that alternative signaling pathways, such as BMP signaling, may maintain partial osteogenic capacity even when canonical Wnt signaling is inhibited. BMP-2 has been shown to induce osteoblast differentiation through a Wnt autocrine loop, and this BMP-driven pathway is not blocked by DKK-1. The incomplete reversal may also reflect the experimental time course; extended treatment or higher antibody concentrations might further improve recovery (9, 44, 45). Although DKK-1 is primarily known as an antagonist of canonical Wnt/β-catenin signaling, it may also indirectly affect non-canonical branches (e.g., Wnt/PCP and Wnt/Ca²^+^ pathways) that signal through RhoA, Rac, and JNK (46, 47). Excessive non-canonical signaling has been implicated in osteoclastogenesis and bone loss (48). Because our study focused exclusively on the canonical axis, we cannot rule out the possibility that part of the anti-resorptive effect observed upon DKK-1 neutralization involves attenuation of aberrant non-canonical signals. Dedicated pathway-specific inhibition or reporter assays would be required to dissect these contributions.

Several limitations should be acknowledged. First, all functional characterization was performed *in vitro*; the *in vivo* efficacy, pharmacokinetics, and safety of the antibody remain to be established. Second, further assessment of anti-drug antibody (ADA) formation in relevant animal models or clinical settings is required. Third, we did not directly compare our antibody with existing Wnt pathway activators such as anti-sclerostin antibodies, nor did we evaluate potential synergistic effects of dual DKK-1/sclerostin inhibition, which represents an attractive future direction. Notably, while donor-to-donor variability is an inherent feature of primary human cell-based assays, the consistent relative trends observed across multiple independent donors in this study support the robustness of our conclusions.

In summary, our findings establish a mechanistic foundation for this humanized anti-DKK-1 antibody as a dual-action candidate capable of simultaneously promoting bone formation and inhibiting bone resorption via the Wnt/β-catenin/OPG axis. Further preclinical and clinical evaluation is warranted to determine its therapeutic potential.

## Conclusion

In this study, we generated a humanized anti-DKK-1 monoclonal antibody (IgG4) with high binding affinity (EC_50_ = 32.8 ng/mL; KD = 2.760 × 10⁻^10^ mol/L) and demonstrated its dual anabolic and anti-resorptive activity through the Wnt/β-catenin/OPG axis. Mechanistically, the antibody restored Wnt/β-catenin signaling in hMSCs, as evidenced by nuclear β-catenin accumulation, GSK-3β phosphorylation, and c-Myc upregulation, which collectively promoted osteogenic differentiation and mineralization. In a human osteoblast–osteoclast Transwell co-culture model, the antibody upregulated OPG secretion, restored the OPG/sRANKL molar ratio, and significantly suppressed osteoclast marker gene expression and TRAP-positive multinucleated cell formation. Additionally, the antibody exhibited favorable developability properties, including thermal stability, freeze-thaw resistance, and low cellular immunogenicity. These findings establish a mechanistic foundation for this antibody as a dual-action therapeutic candidate capable of concurrently promoting bone formation and inhibiting bone resorption, supporting its further preclinical and clinical evaluation for osteoporosis therapy.

## Data Availability

The datasets presented in this study can be found in online repositories. The names of the repository/repositories and accession number(s) can be found in the article/**Supplementary Material**.
