# Supplementary material for: A dual-action humanized DKK-1 antibody that activates the Wnt/β-catenin signaling pathway and inhibits the RANKL signaling pathway for osteoporosis therapy
Source: Front Immunol. 2026 Aug 27;17:1906434. doi: 10.3389/fimmu.2026.1906434 (PMC13557969; doi:10.3389/fimmu.2026.1906434)
Supplement: Supplementary file 1 [file SupplementaryFile1.doc]

**Supplementary Materials**

**Table S1. The information of the primers used in the experiment.**

| **Gene** | **Forward primer (5’→3’)** | **Reverse primer (5’→3’)** |
| --- | --- | --- |
| RPLP0 | AGCCCAGAACACTGGTCTC | ACTCAGGATTTCAATGGTGCC |
| PPIA | GTTTATGTTGTCAGGGTGGTGC | GTATGCTTTAGGATGAAGTTCTC |
| ALP | TCCTGGGCGATGGGATG | CCATGGCCAGGGGTATCT |
| Axin2 | AGGCCCTGCTGTAAAAGAGAG | AGTTCCTCTCAGCAATCGGC |
| BGLAP | ATGAGAGCCCTCACACTCCT | CTTGGACACAAAGGCTGCAC |
| COL1A1 | GAGGGCCAAGACGAAGACATC | CAGATCACGTCATCGCACAAC |
| RUNX2 | TCGCCTCACAAACAACCACA | GCTTGCAGCCTTAAATGACTCT |
| SP7 | GCCACACCAACACACTTTCTA | CTGAGGGACAGCAGGAAATAAG |
| ACP5 | GTGACCCGGGGAGATCTGTG | CTGAGCCTTTATTCCCTCCCTG |
| CALCR | ATTCCCACTGATCCACCCAC | AAGAATTGGGGTTGGGTGATTT |
| CTSK | TTCTGCTGCTACCTGTGGTG | GCCTCAAGGTTATGGATGGA |
| ITGB3 | CTGCCGTGACGAGATTGAGT | ATTAAGTGCCCCGGTACGTG |
| MMP9 | TTTGAGTCCGGTGGACGATG | CGCCAGTACTTCCCATCCTT |
| NFATC1 | AAGTCAGCGGAGGAAGAACA | TGGAGGTCTGAAGGTTGTGG |

RPLP0 and PPIA were used as reference genes for hMSCs and hPBMCs, respectively, based on prior validation studies. Primer sequences were either adopted from previously published reports or designed using Primer‑BLAST (NCBI) and verified by melting curve analysis. ALP, AXIN2, BGLAP, COL1A1, RUNX2, and SP7 are osteogenic marker genes; ACP5, CALCR, CTSK, ITGB3, MMP9, and NFATC1 are osteoclast marker genes.

**Table S2. Monomer content of hDKK‑1 mAb in different formulation buffers after repeated freeze‑thaw cycles, as determined by SEC.**

| Number of freeze‑thaw cycles | Formulation A (%) | Formulation B (%) | Formulation C (%) | Formulation D (%) |
| --- | --- | --- | --- | --- |
| 1 | 97.6 | 97.8 | 98.0 | 97.8 |
| 3 | 97.8 | 97.9 | 97.6 | 98.0 |
| 5 | 97.5 | 98.1 | 97.6 | 97.8 |

Monomer content (%) was determined by size‑exclusion chromatography (SEC) after 1, 3, and 5 freeze‑thaw cycles.

**Table S3.** **Quantification of Alizarin Red S staining by ImageJ analysis**

| Group | Area (pixels²) | Mean | Min | Max | IntDen | **% Area** |
| --- | --- | --- | --- | --- | --- | --- |
| Control | 59,030 | 98.802 | 49 | 115 | 5,832,255 | **53.31** |
| Undiff | 0 | — | — | — | — | **0.00** |
| Wnt3a | 112,349 | 77.450 | 45 | 115 | 8,701,402 | **99.35** |
| DKK‑1 | 19,842 | 100.426 | 72 | 115 | 1,992,643 | **17.89** |
| DKK‑1 mAb | 61,545 | 104.424 | 64 | 115 | 6,426,764 | **56.33** |

Alizarin Red S staining was quantified using ImageJ software (NIH, USA). Images were converted to 8‑bit grayscale, and a uniform threshold (Min = 0, Max = 115) was applied to all images to identify Alizarin Red S‑positive areas. The percentage of positive area (% Area) was calculated for each well. Mean, Min, and Max represent the gray value statistics of the selected thresholded area. IntDen (Integrated Density) is the sum of gray values within the thresholded area. Data are representative of three independent experiments.
